# MIL-101(Fe)-derived porous amorphous materials for efficient Congo red adsorption†

**DOI:** 10.1039/d5ra01603g

**Published:** 2025-05-14

**Authors:** Zhongben Zhou, Changfeng Zeng, Lixiong Zhang, Liang Yu

**Affiliations:** a State Key Laboratory of Materials-Oriented Chemical Engineering, College of Chemical Engineering, Nanjing Tech University Nanjing 211816 China lixzhang@njtech.edu.cn; b College of Mechanical and Power Engineering, Nanjing Tech University Nanjing, 211816 China; c Chemical Technology, Luleå University of Technology SE-971 87 Luleå Sweden liang.yu@ltu.se

## Abstract

In this study, MIL-101(Fe)-derived porous amorphous materials were prepared using 2-methylimidazole (2-MelM) as a competitive ligand, and their adsorption performance for Congo red (CR) was investigated. The characterization of the porous amorphous materials was carried out using various techniques, such as SEM, XRD, BET, FT-IR, and XPS. The effects of the mass ratio of MIL-101(Fe) to 2-MelM and the amorphization time on material properties were studied, and the influences of dye concentration, pH, and temperature on adsorption performance were evaluated. The results showed that amorphization enhanced the adsorption performance. The sample prepared with a mass ratio of MIL-101(Fe) : 2-MelM = 1 : 16 and an amorphization time of 10 min displayed the highest adsorption capacity of 7078 mg g^−1^ under conditions of pH 7, an adsorption time of 24 h, a temperature of 45 °C, and an initial concentration of 600 mg L^−1^, representing a 47% improvement compared to MIL-101(Fe). Monolayer adsorption was confirmed by fitting the adsorption isotherm using the Langmuir equation, and the saturated adsorption capacity was estimated to be 7095 mg g^−1^. This value was remarkably high compared with most reported values in the literature. Reuse experiments indicated high stability of the materials. Various characterizations revealed that hydrogen bonding, electrostatic interactions, and π–π interactions contributed to the adsorption process. This study demonstrated that the amorphization of MIL-101(Fe) efficiently enhanced the CR adsorption capacity.

## Abstract 2

Compared to MIL-101(Fe), amorphized MIL-101(Fe) displayed excellent adsorption capacity for Congo red.

## Graphical abstract

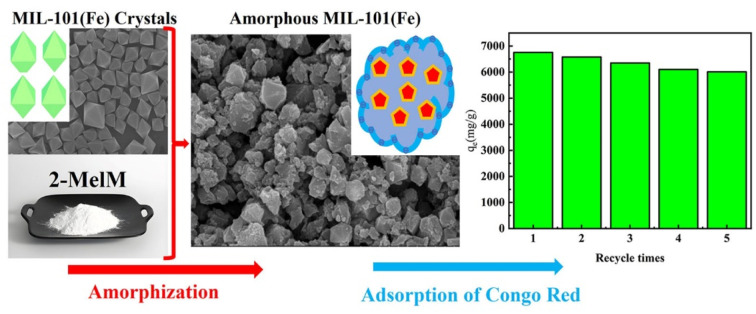

## 1. Introduction

**Scheme 1:**
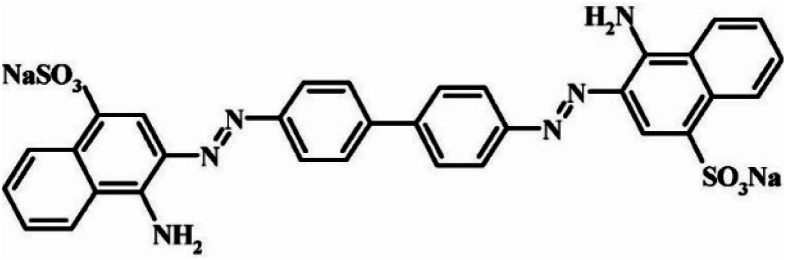
Structural formula of CR.

Among the widely used anionic azo dyes, Congo red (CR) is commonly employed in coloring leather, textiles, plastics, food, cosmetics, papermaking, pharmaceuticals, and other products.^1–3^ It comprises two symmetrical naphthyl groups (each containing one sulfonic acid group and one primary amine group) and a central biphenyl group (Scheme 1).^4^ Consequently, its structure is relatively stable, making the degradation of CR-containing wastewater challenging. The adsorption method, known for its operational simplicity, low cost, and high efficiency, is widely regarded as an excellent treatment approach for removing CR from water.^5,6^ Therefore, it has attracted extensive attention, with the key challenge being the development of high-performance adsorbents.

In the past few decades, researchers have attempted to use inexpensive and readily available materials without the need for processing, such as waste wood,^7^ fungi,^8^ plants,^9^ and other biomass materials, as well as clay minerals like montmorillonite,^9^ kaolin,^10^ and bentonite^11^ for the adsorption of CR. However, these materials often suffer from relatively low porosity, high sensitivity to pH variations, or negatively charged surfaces.^12^ All these factors contribute to the relatively low adsorption capacity of anionic CR, typically not exceeding 400 mg g^−1^. Chitosan, a cationic biopolymer, has also been used to adsorb CR, with reported adsorption capacities of 5107 mg g^−1^ for chitosan nanoparticles^13^ and 44 956 mg g^−1^ for chitosan solution.^14^ However, both forms face challenges with regeneration. Hydrogels, such as polyacrylamide,^15^ polyacrylic acid,^16^ and polyvinyl alcohol,^17^ can be modified through impregnation, cross-linking, or grafting techniques^18^ to enhance porosity and surface charge, thereby significantly improving CR adsorption capacities, reaching up to 2220 mg g^−1^.^19^ However, the adsorption capacities of these materials are still much lower than those of positively charged metal oxide nanomaterials such as MgO,^20^ polyhedral Cu_2_O,^21^ and ZnO.^22^ Nevertheless, due to their small particle size, metal oxide nanomaterials are difficult to recover. To address this issue, some studies have developed mixed metal oxide composites, such as MgO–SiO_2_ composites^23^ and Mg–Al-mixed metal oxides.^24^ These composites can be easily shaped and recovered, while still exhibiting relatively high adsorption capacities of around 4000 mg g^−1^.^23^ Nevertheless, further improvement in adsorption capacity is still needed compared to the most effective adsorbents.

On the other hand, porous materials such as porous carbons and zeolites have been employed for CR adsorption. However, due to negatively charged surfaces and small pore sizes, zeolites have limited capacity for absorbing anionic dyes like CR.^25^ Carbon nanotubes and nanofibers, which serve as efficient adsorbents, are prone to agglomeration, which hides active adsorption sites and limits their efficiency.^25^ Their CR adsorption capacities are thus similar to those of the aforementioned clay mineral. Porous composites prepared from carbon nanomaterials, such as amorphous carbon nanotubes (ACNT), and clay minerals, such as MgAlF_5_·1.5H_2_O/ACNT composites (MAFH/ACNT),^26^ have improved CR adsorption capacity from 468 to 4261 mg g^−1^. However, their use of expensive carbon nanotubes and rare volcanic fumarolic minerals, combined with a complex synthetic process, limits their practical application. Over the past decade, metal–organic framework (MOF) materials including MIL-100(Fe),^27^ ZIF-67,^28^ and UIO-67 (ref. 29) have been investigated for CR adsorption due to their unique porous structures and large surface areas. However, perhaps due to weak interactions between their surface and CR molecules, their adsorption capacities for CR are comparable to those of metal oxides. Several modifications have been explored to enhance MOF adsorption performance toward CR. For instance, incorporating Co and Fe into bimetallic Co/Fe-BDC-(1),^30^ increased the CR loading from 775 mg g^−1^ to 1936 mg g^−1^, ascribed to defects formation resulting from metal heterogeneity. The presence of defects also generates more active sites. Introducing urea into UiO-67 introduces nitrogen-containing functional groups, improving interaction with sulfonic acid groups in CR and increasing adsorption from 1237 to 2360 mg g^−1^.^31^ Converting MOF-1 into an amorphous form (aMOF-1) leads to structural changes and defect exposure, enhancing adsorption from 870 mg g^−1^ to 2417 mg g^−1^.^32^ Nevertheless, the MOF-based adsorbents still demonstrate moderate adsorption capacities, and post-treatment strategies, such as amorphization, offer a promising route for improvement.

In this study, 2-MelM was used as a competitive ligand to derive MIL-101(Fe) into a porous amorphous material, aMIL-101(Fe) (aM), for the adsorption of CR. As a member of the MIL series of MOFs, MIL-101(Fe) has been recognized as a promising adsorbent. Its unique structural stability, versatile surface functionality, and high surface area provide excellent adsorption capacity and selectivity for removing organic pollutants from various water environments.^33^ Furthermore, the unsaturated Lewis acid sites in the framework could enhance the adsorption ability through electrostatic interactions.^33^ Based on these advantages, MIL-101 (Fe) has been used to adsorb various dyes,^34–36^ including CR,^37^ but with limited adsorption capacity, typically lower than 100 mg g^−1^. In this study, we, for the first time, improved the adsorption capacity of MIL-101(Fe) by applying an amorphizing treatment. We studied the amorphization process and characterized the resulting amorphous material using XRD, SEM, BET, FT-IR, and XPS. The amorphization and adsorption conditions were investigated thoroughly. To study the adsorption kinetics, the pseudo-first-order and pseudo-second-order kinetic models were applied, and the Langmuir, Freundlich, and Dubinin–Radushkevich (DR) models were used to describe the adsorption isotherms obtained at 45 °C. The adsorption mechanism was analyzed based on the adsorption and modeling outcomes.

## 2. Experimental

### 2.1 Amorphization of MIL-101(Fe)

MIL-101(Fe)-derived porous amorphous materials were synthesized by amorphizing MIL-101(Fe) using 2-methylimidazole (2-MelM). This method was inspired by the reported amorphization of MIL88B using 2-MelM.^38^ MIL-101(Fe) was synthesized based on a previously reported method.^39^ Briefly, 60 mL *N*,*N*-dimethylformamide (DMF) was used to dissolve 2.7 g FeCl_3_·6H_2_O and 0.82 g terephthalic acid, and the mixture was stirred for 1 h at 20 °C. The mixture was poured into an autoclave with a poly(tetrafluoroethylene) liner and heated at 110 °C for 20 hours. The MIL-101(Fe) products were obtained by centrifuging and washed with DMF and anhydrous ethanol. The products were then dried at 70 °C overnight and activated at 150 °C for 10 h under vacuum. Amorphization was carried out by ultrasonically dispersing 0.1 g of MIL-101(Fe) in 10 mL of methanol, followed by the addition of 20 mL of 2-MelM aqueous solution (0.08 g mL^−1^). The suspension was left to stand still for 10 minutes and then subjected to centrifugation. The mass ration of MIL-101(Fe) to 2-MelM was 1 : 16. The resulting solids were washed with distilled water and dried at 80 °C for 12 h to yield MIL-101(Fe)-derived porous amorphous materials, referred to as aM-2. To study the effect of the MIL-101(Fe) to 2-MelM mass ratio, 20 mL of 2-MelM aqueous solution at concentrations of 0.075 g mL^−1^ and 0.085 g mL^−1^ were used, resulting in mass ratios of 1 : 15 and 1 : 17, respectively. The corresponding samples were named as aM-1 and aM-3.

### 2.2 Material characterizations

Philips Quanta 200 and Hitachi S4800 scanning electron microscopes (SEM) were used to observe the morphologies and microstructures. The FT-IR (Fourier Transform Infrared Spectroscopy) spectra of the samples were acquired with a NICOLET, NEXUS 670 instrument. Samples were mixed with KBr powder (mass ratio 1 : 10) and prepared to be pelleted for analysis. The scanning covered the range from 4000 cm^−1^ to 500 cm^−1^ with a resolution of 2 cm^−1^. Bruker D8-Advance X-ray diffractometer was used to measure XRD patterns. The X-ray radiation source was Cu Kα, operating at a voltage of 40 kV and a current of 36 mA. The pore structures were characterized by nitrogen adsorption and desorption (BELSORP II). The degassing conditions were 150 °C for 10 h under vacuum. The calculation of the total pore volume was based on the nitrogen adsorption quantity when the relative pressure was 0.99. We used the Brunauer–Emmett–Teller (BET) method and the adsorption curve in the relative pressure range of 0.05–0.25 to calculate the surface area (*S*_BET_) and used the Barrett–Joyner–Halenda (BJH) method to calculate the mean pore size of mesopores. Zeta potentiometer (MALVERN 3000) was used to test the sample potential. Prior to testing, the sample was dispersed in water using ultrasonic treatment. The changes in the surface elements of Fe, C, and O were studied by AXIS SUPRA X-ray photoelectron spectroscopy (XPS).

### 2.3 Adsorption studies

Typical adsorption CR experiments were conducted by dispersing 3 mg of the MIL-101(Fe)-derived porous amorphous material in 200 mL of CR aqueous solution at various concentrations (100–600 mg L^−1^) at specified temperatures. To investigate the effect of pH on adsorption, 0.1 M HCl and 0.1 M NaOH solutions were used to adjust the pH. For adsorption capacity analysis, 2 mL of CR aqueous solution was taken using a syringe connected with a 0.2 μm filter during adsorption, and the remaining CR concentration was detected using a Shimadzu UV-2450 UV-Visible spectrophotometer (wavelength 497 nm). The solution was diluted with distilled water before spectrophotometric analysis.

Eqn (1) and (2) were employed to compute the adsorption capacity of CR.1
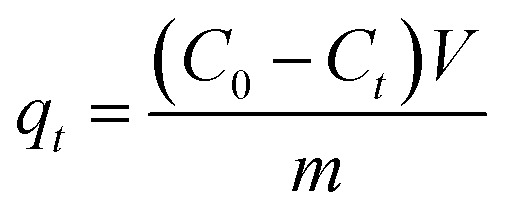
2
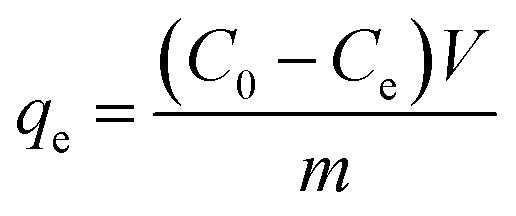


In these equations, *C*_0_, *C*_e_, and *C*_*t*_ represent the CR concentration at initial, at equilibrium, and at adsorption time *t* (mg L^−1^), respectively. *q*_*t*_ represents the adsorption amount at the adsorption time *t*, while *q*_e_ stands for the adsorption amount at equilibrium. *m* refers to the mass (measured in grams) of the adsorbent employed in the experiment, and *V* indicates the CR aqueous solution volume (in liters).

Optimization of the adsorption experimental variables was also considered important for understanding the relative significance of different adsorption parameters.^40,41^ In this study, response surface methodology was employed to predict the optimal adsorption experimental conditions, following a previously reported procedure.^41^ More details are provided in the ESI.†

### 2.4 Isotherm studies

The adsorption isotherms were analysed using the linear forms of the Langmuir and Freundlich equations to investigate the adsorption mechanism and determine the isotherm type. The corresponding isotherm plots were generated to visualise the adsorption behavior. The linear equations of these two are presented in eqn (3) and (4) below.

Langmuir equation:3
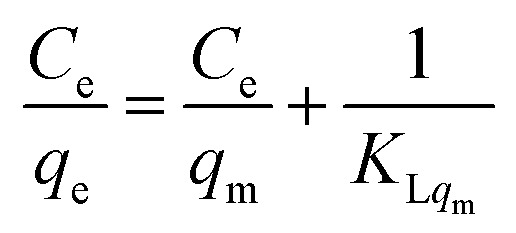


Freundlich equation:4
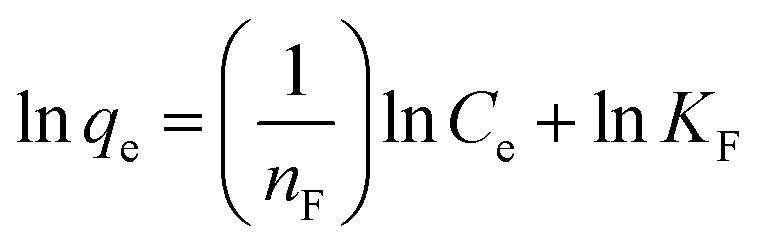


Among them, *C*_e_ represents the equilibrium concentration, *q*_m_ is the monolayer saturation adsorption amount, *q*_e_ is the equilibrium adsorption amount, *K*_L_ represents the Langmuir equilibrium constant, and *n*_F_ and *K*_F_ represent constants regarding the adsorption strength and adsorption amount.

We also used the Dubinin–Radushkevich (DR) model^42^ to fit the adsorption data. The DR model is defined by:

5 ln *q*_e_ = ln *q*_d_ − *K*_T_*ε*^2^

6
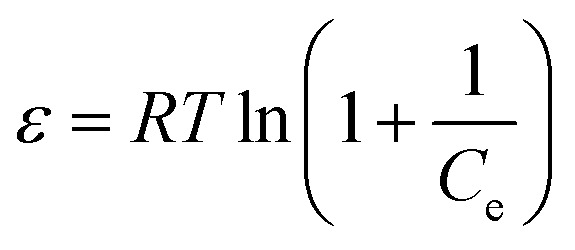
7
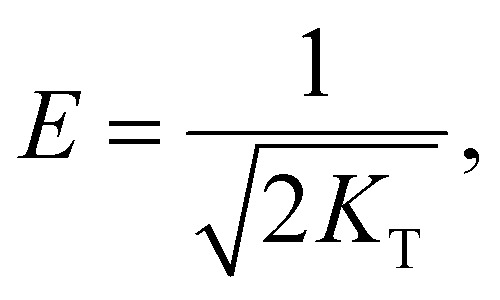
where *q*_d_ is the maximum adsorption capacity, *K*_T_ is a constant related to adsorption energy, *R* is the gas constant (8.314 J (mol^−1^ K)), *T* is the absolute temperature (K), *E* is the energy of adsorption (kJ mol^−1^) and *C*_e_ is the adsorption equilibrium concentration (mg L^−1^).

### 2.5 Kinetic studies

Adsorption kinetic models (pseudo-first-order and second-order) were employed to fit the adsorption results and investigate the adsorption mechanism. The corresponding equations of the two are shown in eqs (8) and (9) below.

First-order dynamic:8
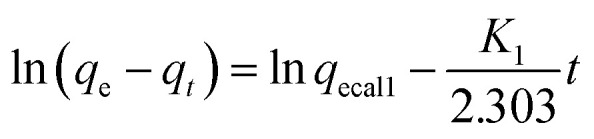


Second-order dynamic:9
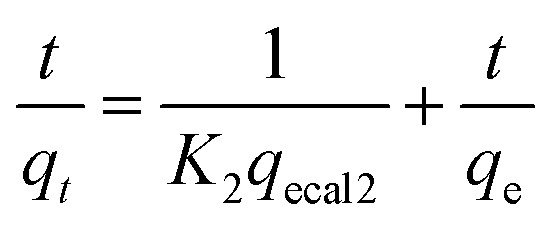


Among them, *q*_ecal1_ and *q*_ecal2_ respectively represent the equilibrium adsorption amounts calculated and simulated by the pseudo-first-order and second-order equations, and *K*_1_ and *K*_2_ represent the pseudo-first-order and second-order kinetic rate constants, respectively.

The chi-square *χ*^2^ between the experimental data and the calculated values is evaluated using the following equation.^40,43^10
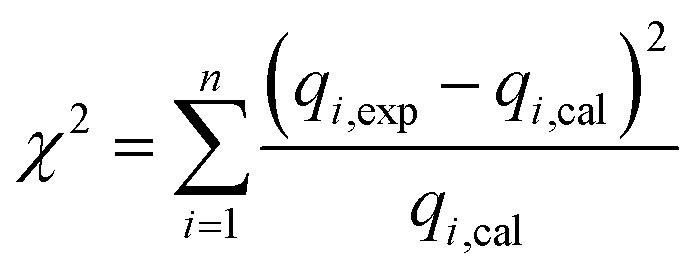


In this equation, *q*_*i*,exp_ and *q*_*i*,cal_ are the experimental adsorption capacity and the calculated adsorption capacity (mg g^−1^), and *n* is the number of measurements.

### 2.6 Regeneration of adsorbent

After adsorption, the used adsorbents were recovered from the aqueous solution by centrifugation and regenerated by dispersing them in methanol (*ca.* 100 mL) followed by ultrasonic treatment for 30 min. The regeneration steps were repeated several times until a colorless methanol supernatant was observed. The regenerated adsorbent was reused after rinsing with deionized water, drying at 70 °C, and activating at 80 °C under vacuum 10 h.

## 3. Results and discussion

### 3.1 Preparation of materials and structural study

**Fig. 1:**
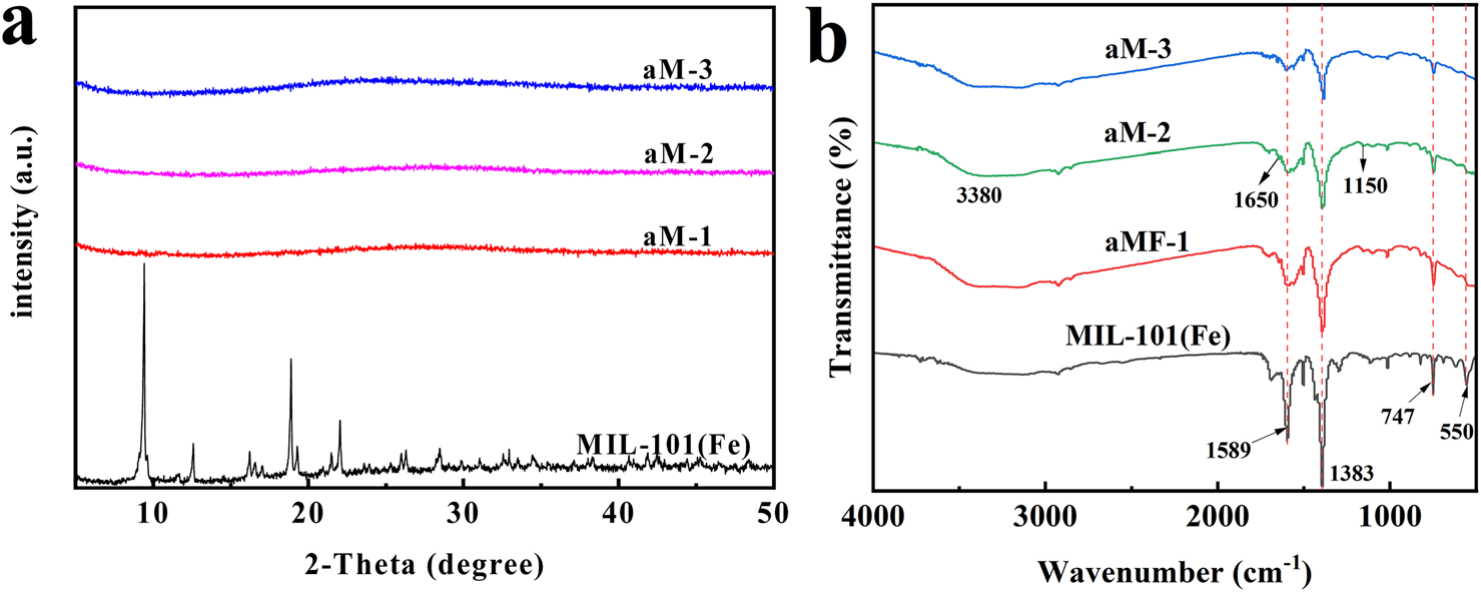
(a) XRD patterns and (b) FT-IR spectra of aM-1, aM-2, aM-3, and MIL-101(Fe).

MIL-101(Fe)-derived porous amorphous materials were prepared by adding 2-MelM to a mixture of orange MIL-101(Fe) particles and methanol. Brown precipitation gradually formed in the uniform orange solution. As the amount of 2-MelM increased, the color of the flocculent gradually darkened. The XRD patterns of MIL-101(Fe) and aM-1, aM-2, and aM-3 are illustrated in Fig. 1a. The diffraction peaks at 9.36°, 12.69°, 16.32°, 19.36°, and 22.16° indicated the successful formation of MIL-101(Fe) with high crystallinity.^44^ No diffraction peaks were observed in the aM-1 to aM-3 samples, suggesting the destruction of the crystal structure and the formation of amorphous structures after the addition of 2-MelM to MIL-101(Fe).

The FTIR spectra of MIL-101(Fe) and aM-1, aM-2, and aM-3 are illustrated in Fig. 1b. The specific absorption bands at 550, 747, 1383, and 1589 cm^−1^ in the synthesized MIL-101(Fe) correspond to the Fe–O vibrational modes, the C–H bending vibrations of the benzene ring in terephthalic acid, and the asymmetric and symmetric stretching vibrations of the carboxylate groups, respectively. The broad band around 3020–3630 cm^−1^ was attributed to the water present in the material.^45^ These absorption bands matched those observed for standard MIL-101(Fe) in the literature,^45^ further confirming the successful synthesis of the samples. These absorption bands were also present in aM-1, aM-2, and aM-3. However, the peak intensities at 1589, 1383, 747, and 523 cm^−1^ gradually decreased with increasing 2-MelM content, while the broad band around 3020–3630 cm^−1^ became more intense. This indicated that part of the terephthalic acid was released from the samples and replaced by –OH groups. In addition, a new absorption band detected at 1650 cm^−1^ corresponded to the Fe–O vibration mode in FeOOH, suggesting the formation of FeOOH.

**Fig. 2:**
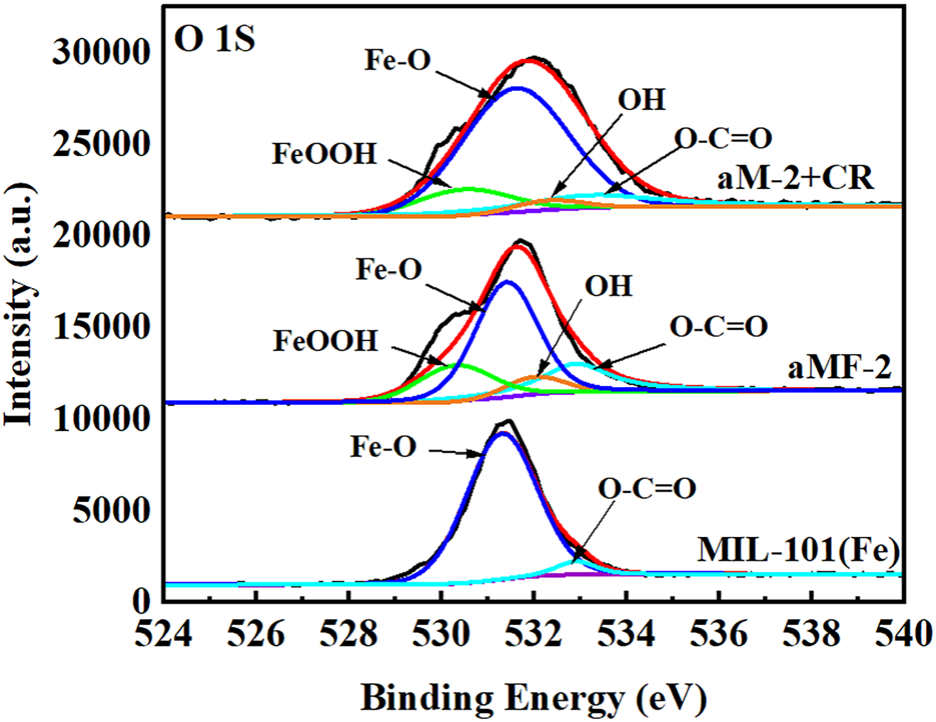
High-resolution XPS spectra of O 1s for MIL-101(Fe) and aM-2 before and after adsorbing CR.

Fig. 2 illustrates the high-resolution XPS O 1s spectra of MIL-101(Fe). The O 1s spectrum of MIL-101(Fe) was fitted to the carboxylate bond (533 eV) and the Fe–O bond (531 eV).^45^ For aM-2, two peaks observed at 532 eV and 530 eV corresponded to the –OH group and lattice oxygen of Fe–OOH,^38^ respectively, indicating the formation of FeOOH and additional –OH groups during the amorphization process. This result was consistent with the FTIR analysis described above. Based on the XPS elemental content analysis data (Table 1), significant changes occurred in the contents of Fe, C, and O elements before and after the amorphization process. Specifically, the O and Fe contents in aM-2 increased from 25.3% to 32.0% (O) and from 5.9% to 9.4% (Fe), while the C content decreased from 68.8% to 58.6%.

**Table 1:** C, O, and Fe contents calculated from XPS analysis

Sample	C (at%)	O (at%)	Fe (at%)
MIL-101(Fe)	68.8	25.3	5.9
aM-1	61.6	29.9	8.5
aM-2	58.6	32.0	9.4
aM-3	59.7	30.1	10.2

**Fig. 3:**
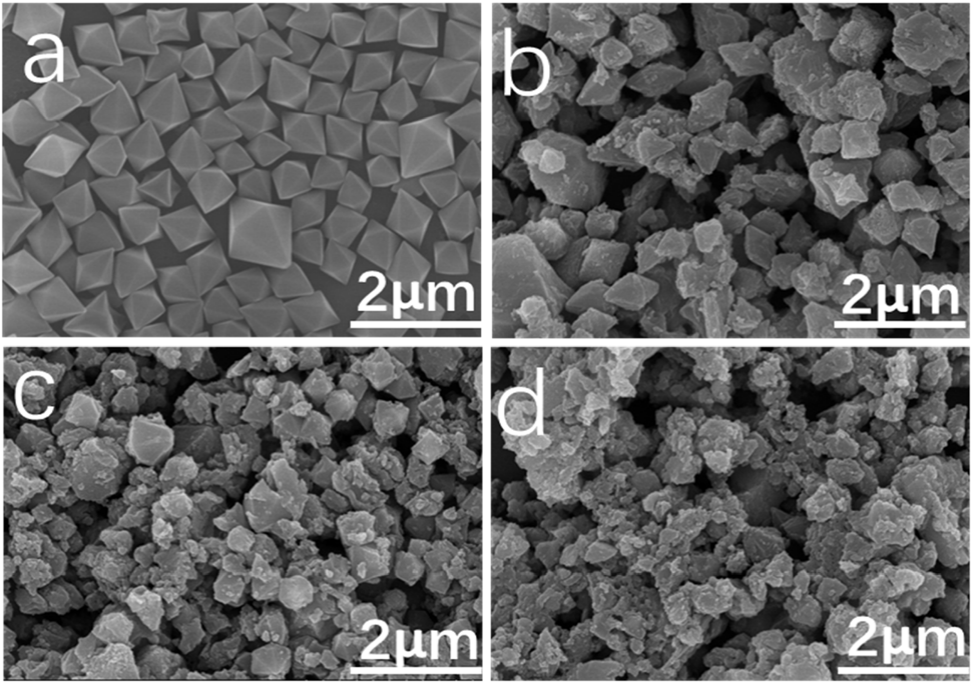
SEM images of MIL-101 (Fe) (a), aM-1 (b), aM-2 (c), and aM-3 (d).

Fig. 3 displays the SEM images of MIL-101(Fe) and aM-1, aM-2, and aM-3. The pristine MIL-101(Fe) crystals exhibited a regular octahedral shape, with a smooth surface and well-distributed particles. The average particle size was approximately 500 nm, consistent with MIL-101(Fe) prepared using a similar method reported in the literature.^39^ A noticeable change occurred after amorphization, with the shape transitioning from regular octahedra to irregular morphologies. The particle size decreased, and aggregation was observed as the amount of 2-MelM increased during the amorphization process.

**Fig. 4:**
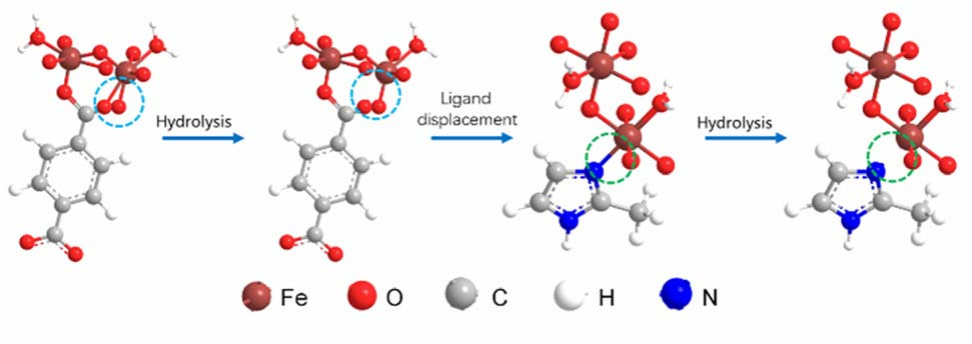
Scheme of the ligand-competition process for MIL-101(Fe) amorphization (only the atoms in the framework close to the reaction center are shown).

Based on our experimental observations, a schematic of the ligand-competition amorphization process was created and is shown in Fig. 4. In this process, a six-coordinated Fe atom in the MIL-101(Fe) framework was hydrolyzed to form a five-coordinated Fe atom. Subsequently, 2-MelM replaced the terephthalic acid ligand in the framework, resulting in a metastable structure. This structure was further hydrolyzed, leading to the removal of 2-MelM from the framework and the formation of an amorphous structure, thereby destroying the long-range crystallinity of MIL-101(Fe).

**Fig. 5:**
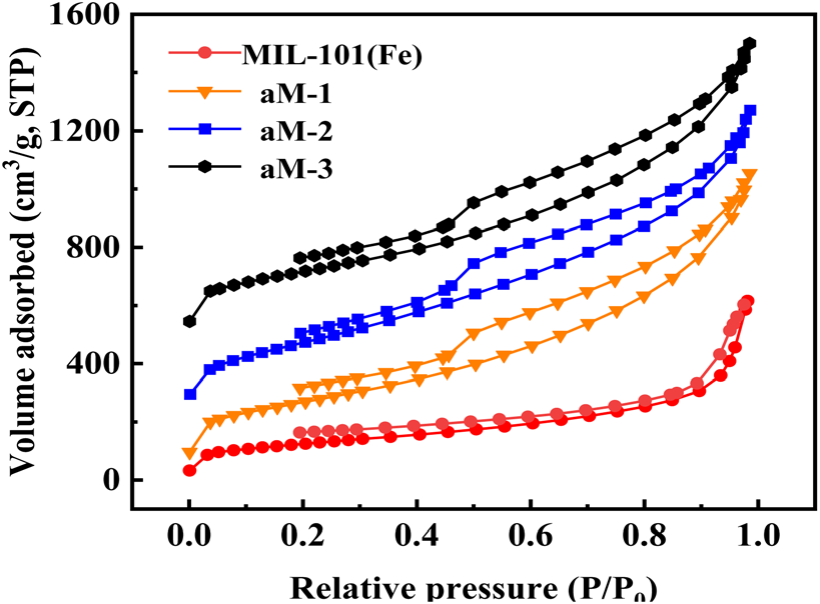
Nitrogen adsorption and desorption isotherms for MIL-101 (Fe), aM-1, aM-2, and aM-3.

Fig. 5 exhibits the nitrogen adsorption and desorption isotherms for MIL-101(Fe), aM-1, aM-2, and aM-3. A type I adsorption–desorption isotherm was observed for MIL-101(Fe), which was consistent with results reported in the literature.^39^ It has also been reported that MIL-101(Fe) exhibits both micropores and mesopores.^33,46^ Our pore size analysis of MIL-101(Fe) confirmed a mesoporous structure with a pore size of about 2.2 nm. After amorphization, type IV isotherms were observed, indicating the formation of a mesoporous structure in the resulting materials. Similar hysteresis loops on the isotherms of aM-1 to aM-3 appeared at nearly identical relative pressures (*p*/*p*_0_ > 0.45), suggesting comparable mesoporous structures among the amorphized samples. The textural properties, including specific surface area, pore size, and pore volume of MIL-101(Fe), aM-1, aM-2, and aM-3 are presented in Table 2. The results showed that the amorphized samples had significantly larger surface areas, pore sizes, and pore volumes, with values for aM-1, aM-2, and aM-3 being very similar.

**Table 2:** Textural properties of MIL-101(Fe), aM-1, aM-2, and aM-3

Sample	BET specific surface area (m^2^ g^−1^)	Total pore volume (cm^3^ g^−1^)	Mesopore size (nm)
MIL-101(Fe)	600	1.3	2.2
aM-1	1314	2.0	3.4
aM-2	1402	2.3	3.7
aM-3	1356	2.2	3.7

### 3.2 CR adsorption

**Fig. 6:**
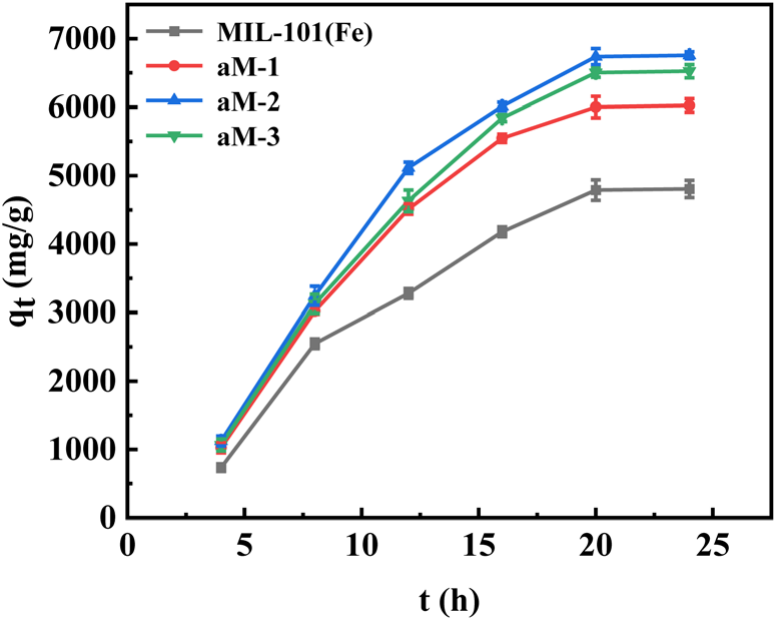
Adsorption loading of CR as a function of adsorption time on different adsorbents at 25 °C. Experimental conditions: 3 mg of aM-2, 120 mL of 200 mg per L CR solution.

The observed adsorption results for MIL-101(Fe), aM-1, aM-2, and aM-3 are presented in Fig. 6. The highest adsorption capacity observed for MIL-101(Fe) was 4806 mg g^−1^. However, significantly higher adsorption capacities of 6025, 6755, and 6523 mg g^−1^ were observed for aM-1, aM-2, and aM-3, respectively. The adsorption capacity for CR improved significantly after amorphization, likely due to changes in pore structures and surface properties. Among the amorphized samples, aM-2 showed the highest adsorption capacity for CR. A crucial aspect contributing to the remarkable adsorption performance of aM-2 was its highly porous nature, characterized by a large total pore volume and surface area. Moreover, its pore dimensions were well-suited to accommodate CR molecules, which measured approximately 2.56 nm × 0.73 nm. This congruence between the pore characteristics and the molecular size of CR is crucial for enabling the high adsorption propensity of aM-2.^30^

**Fig. 7:**
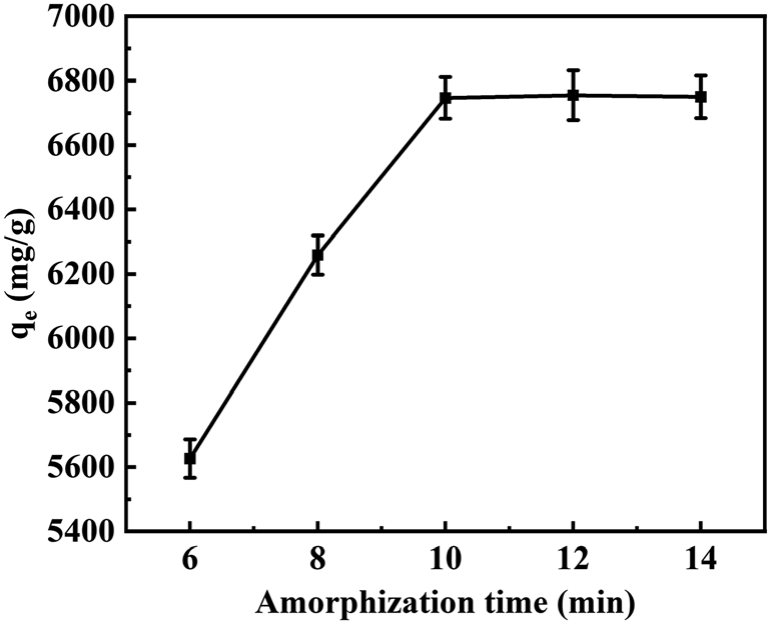
Adsorption loading of CR as a function of amorphization time at 25 °C. Experimental conditions: 3 mg of aM-2, 120 mL of 200 mg per L CR solution, pH 7, adsorption for 24 h.

The impact of amorphization time on sample aM-2 was examined, and the results are depicted in Fig. 7. At an amorphization time of 6 min, a CR adsorption capacity of 5626 mg g^−1^ was observed. When the time was extended to 10 min, the adsorption capacity significantly increased to 6755 mg g^−1^. However, further increases in amorphization time resulted in only marginal changes in adsorption capacity. Based on these findings, the amorphization time was fixed at 10 min for subsequent experiments.

**Fig. 8:**
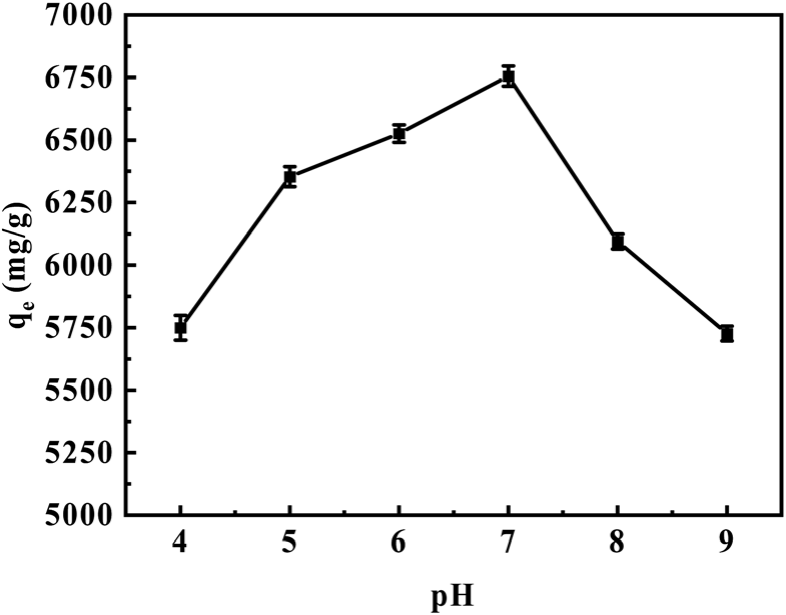
Adsorption loading of CR as a function of solution pH at 25 °C. Experimental conditions: 3 mg of aM-2, 120 mL of 200 mg per L CR solution, adsorption time of 24 h.

Fig. 8 illustrates the adsorption capacity of aM-2 for CR in a 200 mg per L aqueous solution at different pH levels. As the pH increased from 4 to 7, the adsorption capacity significantly improved from 5749 to 6755 mg g^−1^. However, the adsorption capacity slightly decreased to 5726 mg g^−1^ when the pH was further increased to 9.

**Fig. 9:**
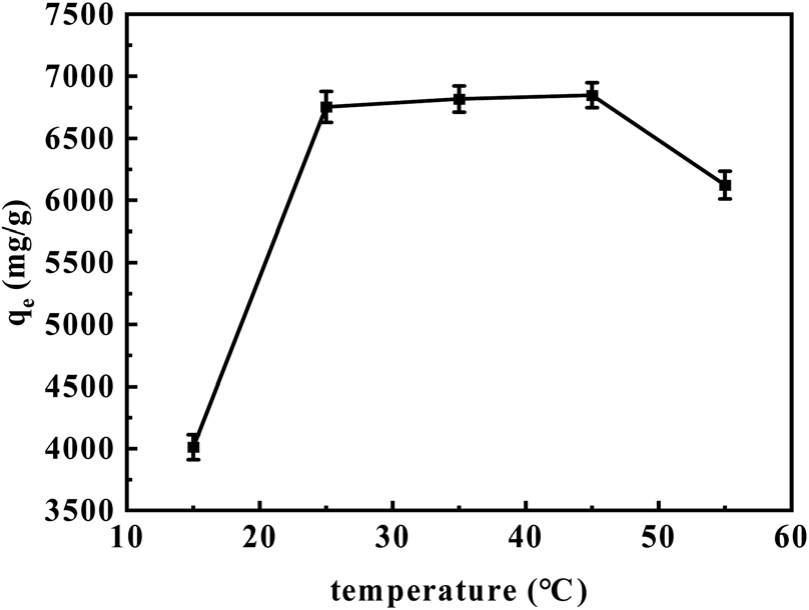
Adsorption loading of CR as a function of solution temperature. Experimental conditions: 3 mg of aM-2, 120 mL of 200 mg per L CR solution, pH 7, adsorption time of 24 h.

Fig. 9 demonstrates the effect of temperature on the adsorption capacity of aM-2. The adsorption capacity improved as the solution temperature was increased from 15 to 45 °C. However, a decreasing trend was observed when the temperature was further increased from 45 to 55 °C. This behavior can likely be attributed to the enhanced mobility of dye molecules at elevated temperatures (15–45 °C) and potential degradation or deactivation of binding sites at temperatures above 45 °C.^47^

### 3.3 Adsorption models

The adsorption isotherm of aM-2 for CR at 45 °C was obtained using CR aqueous solutions with concentrations ranging from 100 to 600 mg L^−1^ at pH 7. As expected, higher adsorption capacities were observed at higher CR concentrations. The highest adsorption capacity, 7078 mg g^−1^, was observed at a CR concentration of 600 mg L^−1^. This value is relatively high compared to many reported materials in the literature, such as hydrogel-based materials and MOF-derived adsorbents, as shown in Table 3. In addition, the experimentally determined optimum adsorption conditions in this study differed slightly from those predicted by the response surface methodology, as shown in Table S1.†

**Table 3:** Typical performance of different adsorbents for CR adsorption

Adsorbent	Initial concentration of CR solution (mg L^−1^)	Maximum adsorption amount (mg g^−1^)	References
Chitosan solution	2000	44 956	14
ZIF-67	100	3900	28
CS nanoparticles	1500	5107	13
MgO	25	136	20
Co/Fe-MOF-(1)	2000	1936	30
MgO/SiO_2_	300	4000	23
MAFH/ACNT	600	4261	26
N-containing UiO-67	2500	1986	31
aMOF-1	1600	2417	32
aM-2	600	7078	This work

**Fig. 10:**
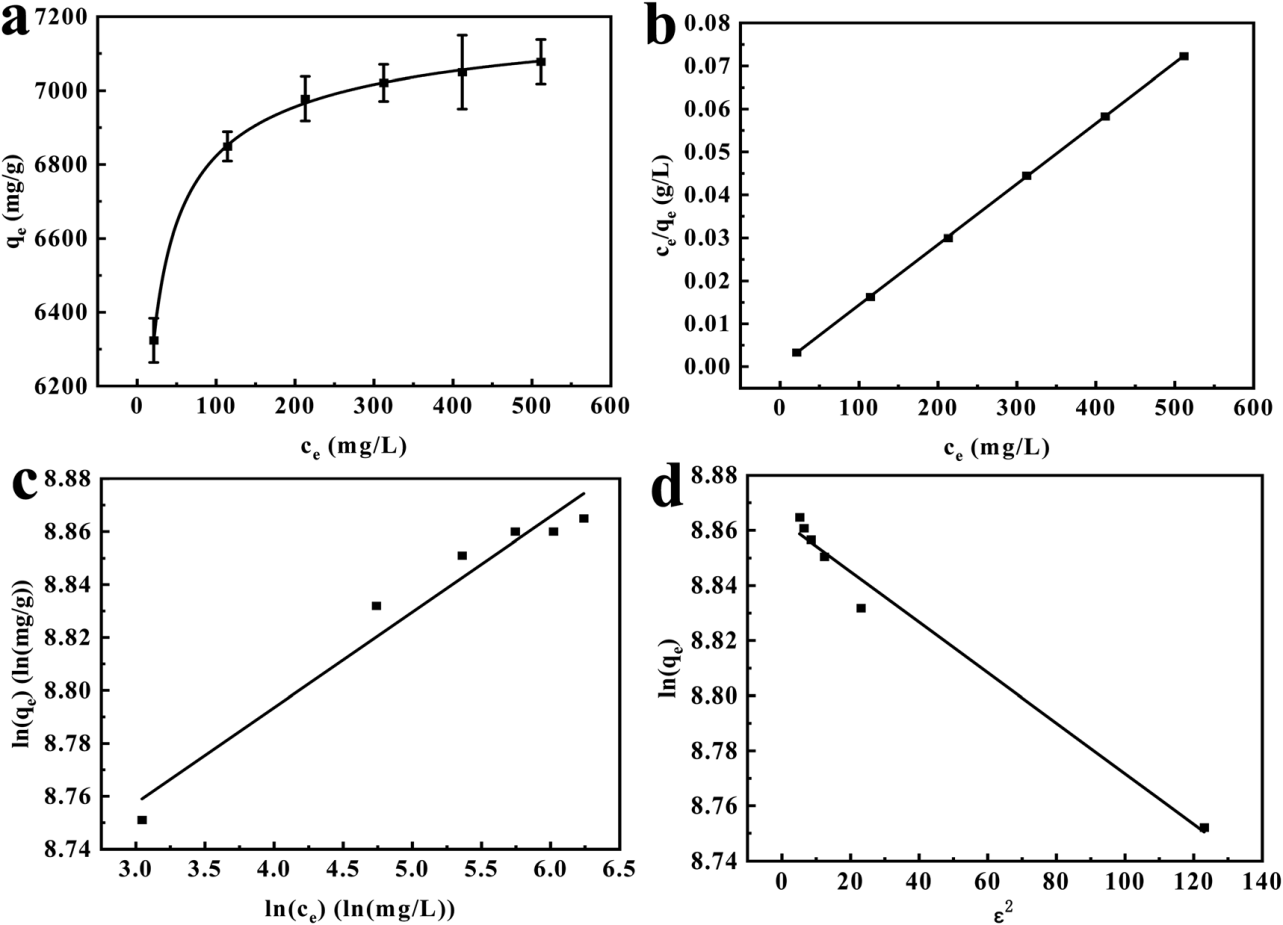
Adsorption isotherm for CR at 45 °C (a), Langmuir fitting result (b), Freundlich fitting result (c), and Dubinin–Radushkevich fitting result (d). Experimental conditions: 3 mg of aM-2, 240 mL of CR solution, 45 °C, pH = 7, adsorption time of 24 h.

The results in Fig. 10 elucidate the linear regression analyses of the Langmuir and Freundlich models applied to the adsorption isotherms. Table 4 enumerates the relevant fitting parameters. The Langmuir model demonstrates a more pronounced ability to describe the adsorption isotherm, as indicated by its substantially higher *R*^2^ value and lower *χ*^2^ value, as shown in Table 4. Based on these results, it could be deduced that the adsorption process on the surface of MIL-101(Fe)-derived porous amorphous materials followed a monolayer adsorption mechanism. The estimated energy of adsorption using the Dubinin–Radushkevich model (Fig. 10d) was about 23.3 kJ mol^−1^, which was higher than the energy typically associated with physical adsorption.^43^ Therefore, the highest adsorption capacity for CR was estimated using the Langmuir equation, yielding a high value of 7095 mg g^−1^. The maximum adsorption capacity estimated from the DR model was 7044 mg g^−1^.

**Table 4:** Key parameters obtained by fitting the Langmuir, Freundlich, and D–R equations to the adsorption isotherm of CR on aM-2

Langmuir		Freundlich		D–R	
*K*_L_ (L mg^−1^)	0.559	*K*_F_ (kJ mol^−1^)	5704.5	*K*_T_ (mol^2^ kJ^−2^)	9.1 × 10^−4^
*q*_m_ (mg g^−1^)	7095	1/*n*	0.0361	*q*_d_ (mg g^−1^)	7044
*R* ^2^	0.999	*R* ^2^	0.956	*R* ^2^	0.978
*χ* ^2^	0.041	*χ* ^2^	0.894	*χ* ^2^	0.164
				*E* (kJ mol^−1^)	24.3

**Fig. 11:**
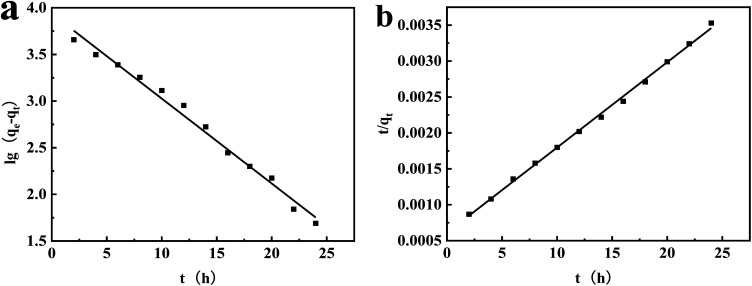
Pseudo-first-order (a) and pseudo-second-order (b) kinetic model linear regression of CR adsorption on aM-2.

Fig. 11 presents the outcomes of the adsorption kinetic fitting, which was performed using the linear forms of the pseudo-first-order and pseudo-second-order models. The fitting constants are listed in Table 5.

**Table 5:** Fitting constants from the pseudo-first-order and pseudo-second-order models for CR adsorption on aM-2

*C*_0_, mg L^−1^	*q*_e_, g g^−1^	Pseudo-first-order model				Pseudo-second-order model			
		*q*_ecal1_, g g^−1^	*K*_1_, min^−1^	*R* ^2^	*χ* ^2^	*q*_ecal2_, g g^−1^	*K*_2_, g (g min)^−1^	*R* ^2^	*χ* ^2^
200	6850	8648	0.321	0.988	3738	7143	3.8 × 10−5	0.998	12.02

The *R*^2^ value of 0.998 was obtained for the pseudo-second-order model, which was significantly superior to that of the pseudo-first-order model. Meanwhile, the calculated equilibrium adsorption capacity (*q*_e_) for CR from the pseudo-second-order model was 7143 mg g^−1^, which was closer to the experimental value of 7078 mg g^−1^. The discrepancy probably resulted from the effect of intra-particle diffusion during the adsorption process.^48^ The estimated *χ*^2^ value from the pseudo-second-order model was also much smaller compared to that from the pseudo-first-order model. These results suggested that the adsorption process could be better described by the pseudo-second-order model, indicating that the adsorption was more likely to be chemisorption.^31^ This would generally lead to monolayer adsorption, which was consistent with the conclusions drawn from the Langmuir and DR modeling results.

### 3.4 Adsorbent reuse

**Fig. 12:**
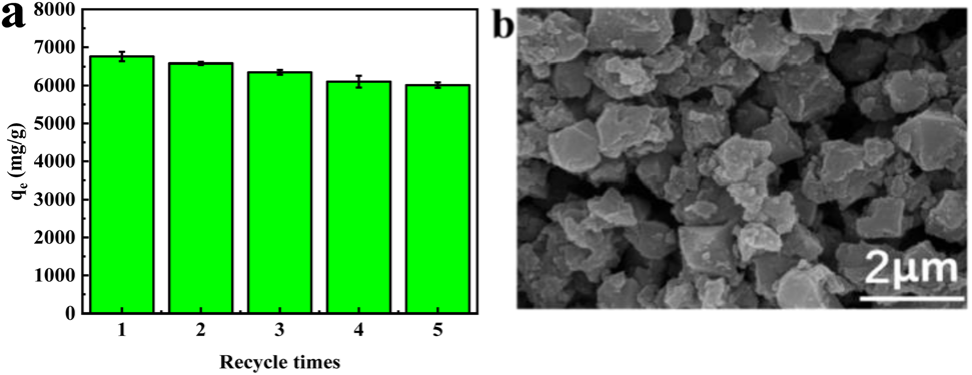
(a) Adsorption capacity at different recycle times; (b) morphology of aM-2 observed by SEM after five cycles. Reuse experiment conditions: 3 mg of regenerated adsorbent dispersed into 120 mL of 200 mg per L CR aqueous solutions at 25 °C and pH 7, adsorption time of 24 h.

The reuse of the adsorbent was examined by adding 3 mg of regenerated aM-2 to 120 mL of a 200 mg per L CR aqueous solution and maintaining it at 20 °C for 24 h. Fig. 12a displays the relationship between adsorption capacity and the number of reuse cycles. The adsorption capacity declined slightly from 6755 to 6011 mg g^−1^ after 5 cycles of reuse, compared to the freshly prepared adsorbent. Moreover, the adsorption capacity in the fourth and fifth cycles were similar. The decline in adsorption capacity was likely due to the presence of strong adsorption sites that could not be regenerated with the currently adopted method. Fig. 12b shows the SEM image of aM-2 after five cycles. The image revealed that the morphology of the sample was preserved, indicating the high stability of the adsorbent.

### 3.5 Adsorption mechanism

**Fig. 13:**
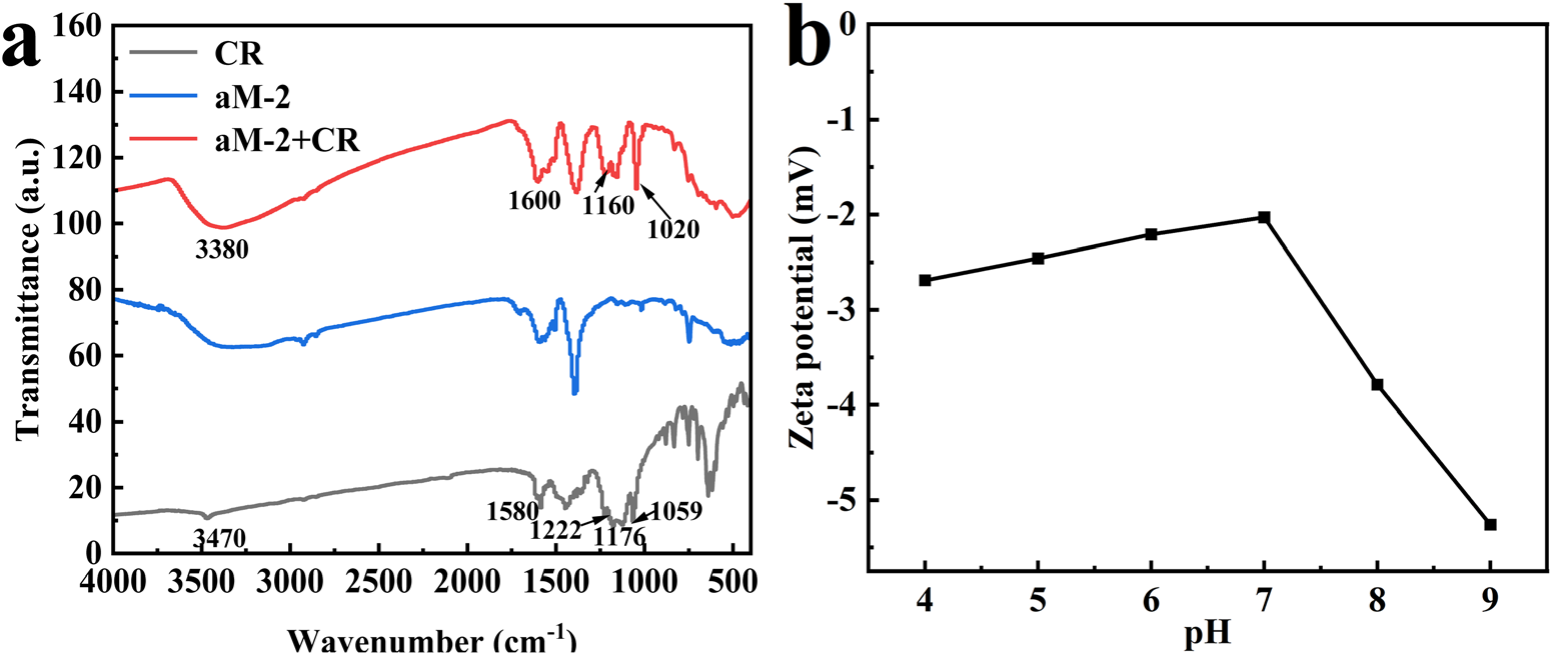
FTIR spectra of CR, aM-2, and aM-2 after adsorption of CR (a), and zeta potential plots of aM-2 at different pH values (b).

Fig. 13a shows the FT-IR spectra of CR, aM-2, and CR loaded aM-2 (aM-2 + CR). After being absorbed by aM-2, some absorption bands in CR molecules were changed. For instance, the absorption band attributed to the –N=N– group in CR shifted from 1580 cm^−1^ to 1600 cm^−1^. The absorption band at 1222 cm^−1^ that was attributed to the S=O stretching vibration in the –SO_3_^−^ group in CR disappeared, and the absorption bands at 1176 and 1059 cm^−1^ that were also attributed to the S=O stretching vibration were shifted to 1160 cm^−1^ and 1020 cm^−1^, respectively.^30^ These changes were consistent with those observed in the bimetallic Co/Fe-MOF after CR adsorption,^30^ and were attributed to hydrogen bonding and π–π interactions between the –N=N– group and the carboxyl group in CR, as well as electrostatic interactions between the –SO_3_^−^ group in CR and Fe^2+^ and Co^2+^. In addition, the stretching vibration of –NH in CR^49^ shifted from 3470 cm^−1^ to 3380 cm^−1^, overlapping with the band ascribed to the –OH group in aM-2. This was likely because hydrogen bonds were formed between the –OH group in aM-2 and the –NH group in CR, consequently increasing the effect of hydrogen bonding in the adsorption process.^45^ This assumption was also verified by the XPS results, as shown in Fig. 2.^38^ The binding energy of the –OH peak in aM-2 increased from 532.2 to 532.4 eV because of the formation of the hydrogen bond; meanwhile, the –OH peak in Fig. 2 became broader.

The adsorption capacity, influenced by pH, indicated that the surface charge exerted a substantial influence on adsorption. Consequently, the zeta potentials of aM-2 in solutions with different pH levels were measured, and the results are presented in Fig. 13b. It was evident that as the pH increased from 4 to 7, the zeta potential of aM-2 increased from −2.69 mV to −2.05 mV. The zeta potential decreased from −2.05 mV to −5.26 mV when the pH further increased from 7 to 9. This trend indicated a negatively charged surface for aM-2, similar to aMOF-1.^32^ Nevertheless, both of them are capable of adsorbing anionic CR. This can be ascribed to the easy adsorption of small Na^+^ ions in CR onto the negatively charged aM-2 surface. Meanwhile, the CR molecules may probably remain intact with Na+, resulting in a high adsorption capacity. The highest adsorption capacity was observed at pH 7, where the surface charge was the least negative. Hence, electrostatic interactions likely played a role in the adsorption mechanism. Although the adsorption mechanisms for CR were, to certain extent, similar to those of other adsorbents, such as bimetallic Co/Fe-MOF^30^ and aMOF-1,^32^ aM-2 exhibited a considerably higher adsorption capacity than both of them and MIL-101(Fe). This was likely due to the larger specific surface area, higher pore volume, suitable pore size, and greater number of adsorption sites in aM-2. Specifically, the BET surface area of aM-2 was as high as 1402 m^2^ g^−1^, far exceeding those of Co/Fe-MOF (4.33 m^2^ g^−1^), aMOF-1 (39.31 m^2^ g^−1^), and MIL-101(Fe) (600 m^2^ g^−1^). The pore diameters of Co/Fe-MOF,^30^ aMOF-1,^32^ and MIL-101(Fe) were 7.38, 2.6, and 2.2 nm, respectively. In comparison to the size of CR molecules (2.56 nm × 0.73 nm), their pore diameters were significantly larger or smaller. The pore diameter of aM-2 was 3.7 nm, which was more suitable for the size of CR molecules and more conducive to the development of a stronger adsorption capacity during the adsorption process. In addition, aM-2 exhibited higher content of O and Fe elements compared to MIL-101(Fe), and more defect sites were formed during amorphization, exposing more functional groups with strong adsorption activities, such as carboxyl, hydroxyl, and Fe^3+^ ions. All these factors contributed to the excellent adsorption performance of aM-2 for CR.

## 4. Conclusion

In conclusion, the present study reports an adsorbent synthesized by amorphizing MIL-101(Fe) for CR adsorption. The amorphization process of MIL-101(Fe) using 2-MelM resulted in significant alterations in its structure, morphology, elemental composition, and properties. Adsorption tests displayed that amorphization of MIL-101(Fe) notably enhanced its adsorption capacity for CR. The influence of several factors, including the mass ratio of MIL-101(Fe) to 2-MelM, amorphization time, CR concentration, pH, and temperature, on the adsorption process were confirmed. Sample aM-2, prepared at a mass ratio of MIL-101(Fe) : 2-MelM = 1 : 16 and an amorphization time of 10 min, exhibited the highest adsorption capacity of 7078 mg g^−1^ under the experimental conditions of pH 7, an adsorption time of 24 h, a temperature of 45 °C, and an initial concentration of 600 mg L^−1^. The Langmuir isotherm model provided the best fit, suggesting monolayer adsorption, with an estimated saturated adsorption capacity of 7095 mg g^−1^. The high heat of adsorption (24.3 kJ mol^−1^), along with the better fit to the pseudo-second-order kinetic model, indicated chemisorption. The adsorption mechanism involved π–π interactions, hydrogen bonding, and electrostatic interactions. The excellent adsorption performance of the MIL-101(Fe)-derived amorphous material was attributed to its large specific surface area, suitable pore size, large pore volume, and the presence of more defect sites and functional groups compared to other materials and pristine MIL-101(Fe). The results of the reuse experiments showed that the adsorption capacity reduced slightly from 6755 to 6011 mg g^−1^ after five adsorption–desorption cycles, demonstrating the high stability of the adsorbent. Overall, the MIL-101(Fe)-derived amorphous material presented in this study exhibits great potential for CR removal from wastewater.

## Data availability

The main text includes all relevant data and methods, and additional data related to this study are available upon reasonable request. Furthermore, the synthesis method of MIL-101(Fe) is supported by the study “A novel magnetic MIL-101(Fe)/TiO_2_ composite for photo degradation of tetracycline under solar light” [https://www.sciencedirect.com/science/article/abs/pii/S0304389418307660]. The amorphization method is supported by the study “Amorphous metal–organic framework-dominated nanocomposites with both compositional and structural heterogeneity for oxygen evolution” [https://onlinelibrary.wiley.com/doi/abs/10.1002/anie.201914587].

## Author contributions

Zhongben Zhou: investigation, formal analysis, writing – original draft, writing – review & editing; Changfeng Zeng: methodology, writing – review & editing; Lixiong Zhang: conceptualization, supervision, funding acquisition, writing – review & editing; Liang Yu: formal analysis, supervision, visualization, writing – review & editing.

## Conflicts of interest

There are no conflicts of interest to declare.

## Supplementary Material

RA-015-D5RA01603G-s001
