# Supplementary material for: MIL-101(Fe)-derived porous amorphous materials for efficient Congo red adsorption
Source: RSC Adv. 2025 May 14;15(20):15940–50. doi: 10.1039/d5ra01603g (PMC12076152; doi:10.1039/d5ra01603g)
Supplement: RA-015-D5RA01603G-s001 [file RA-015-D5RA01603G-s001.pdf]

Supplementary Information (SI) for RSC Advances.
This journal is © The Royal Society of Chemistry 2025

Supplementary information

MIL-101(Fe)-derived porous amorphous materials for efficient Congo Red adsorption

Zhongben Zhou[a], Changfeng Zeng[b], Lixiong Zhang[a]*, Liang Yu[c]*

a. State Key Laboratory of Materials-Oriented Chemical Engineering, College of Chemical Engineering, Nanjing Tech University, Nanjing 211816, China,

b. College of Mechanical and Power Engineering, Nanjing Tech University, Nanjing, 211816, China

c. Chemical Technology, Luleå University of Technology, SE-971 87 Luleå, Sweden.

* E-mail: lixzhang@njtech.edu.cn; liang.yu@ltu.se

In this study, the response surface methodology corresponding to the Box-Behnken design (BBD) in the Design Expert software was employed to carry out the experimental design work to find out the optimium adsorption conditions[1,2]. Specifically, a BBD matrix with three levels and four factors was constructed. The aim was to optimize the selected variables, namely the solution temperature (A), the initial concentration of CR (B), the solution pH (C), and the adsorption time (D), in an effort to achieve the maximum response value. Table S1 clearly shows the range of values for each variable. The experimental operations were carried out in an orderly manner according to this BBD matrix (see Table S1 for details). Subsequently, the obtained response data were fitted into the second-order polynomial model (i.e., The following equation) to establish the internal relationship between the independent variables and the response[3], laying the foundation for subsequent in-depth analysis and precise control.

$$Y = b_0' + \sum_{i=1}^{n} b_i x_i + \sum_{i=1}^{n} b_{ii} x_i^2 + \sum_{i=1}^{n} \sum_{j>1}^{n} b_{ij} x_i x_j$$

Here, Y represents the predicted response value. The regression coefficients corresponding to the quadratic term effect, linear term effect, intercept term effect, and interaction term effect are b_{ii}, b_i, and b_0' respectively, while the independent variables presented in coded units are x_i and x_j. The rationality of the fitted model was examined by using the method of analysis of variance (ANOVA).

Table S1 Box-Behnken design matrix and experimental and predicted responses

Run	A Temperature (℃)	B Concentration (mg/L)	C PH	D Time (h)	Experimental (mg/g)	BBD Predicted (mg/g)
1	40	400	7	14	5719	5434
2	25	400	5	14	5362	4793
3	55	600	7	14	5230	4844
4	40	400	9	4	947	970
5	55	400	9	14	4399	4614
6	55	200	7	14	5061	4843
7	25	400	7	4	1151	1400
8	55	400	5	14	4880	4586
9	55	400	7	24	6298	6550
10	40	400	9	24	5520	5893
11	40	200	9	14	4654	4726
12	40	200	7	4	1108	947
13	40	200	5	14	5161	4916
14	25	400	7	24	6924	6721
15	40	400	7	14	5363	5434
16	40	400	7	14	5363	5434
17	40	400	7	14	5363	5434
18	25	600	7	14	5750	5683
19	55	400	7	4	1048	1205
20	40	600	5	14	5085	5241
21	40	400	7	14	5363	5434
22	25	400	9	14	4833	4637
23	40	600	9	14	4420	4618
24	40	200	7	24	6662	6379
25	25	200	7	14	5426	5479
26	40	400	5	4	1076	1000
27	40	600	7	4	1088	1232
28	40	600	7	24	6542	6515
29	40	400	5	24	6470	6591
30	40	600	7	4	1088	1232

Response surface methodology

The experimental adsorption data were fitted into a quadratic model, a two-factor interaction model, and a linear model respectively.[4] Through the comparative analysis of these statistical parameters, it was found that the quadratic model could fit the experimentally obtained data well. In order to check the applicability of the quadratic

model, an analysis of variance was further carried out, and the results of this analysis of variance have been summarized in Table S2.

The F-value of the model is 243.35, and the corresponding p-value is less than 0.0001, marked as "significant". This indicates that the overall model is significant, meaning that there is a significant linear relationship between the independent variables (A - temperature, B - concentration, C - pH, D - time, etc.) and the response variable in the model. The model can effectively explain the changes in the response variable. The F-value of the lack-of-fit term is 1.26, and the p-value is 0.4438, marked as "not significant". This shows that there is no obvious lack of fit in the model, that is, the model can fit the data well, and there are no important factors or non-linear relationships that are not captured by the model

Table S2. ANOVA for the developed quadratic model.

Source	Sum of Squares	df	Mean Square	F-value	p-value	
Model	1.03E+08	14	7.32E+06	243.35	< 0.0001	significant
A-temperature	5.33E+05	1	5.33E+05	17.73	0.0009	
B-concentration	154.08	1	154.08	0.0051	0.944	
C-PH	8.86E+05	1	8.86E+05	29.45	< 0.0001	
D-time	8.53E+07	1	8.53E+07	2835.34	< 0.0001	
AB	6006.25	1	6006.25	0.1996	0.6619	
AC	576	1	576	0.0191	0.8919	
AD	68382.25	1	68382.25	2.27	0.1539	
BC	6241	1	6241	0.2074	0.6558	
BD	2500	1	2500	0.0831	0.7774	
CD	1.69E+05	1	1.69E+05	5.6	0.0329	
A^2	16744.78	1	16744.78	0.5564	0.468	

B^2	34267.25	1	34267.25	1.14	0.304	
C^2	1.59E+06	1	1.59E+06	52.85	< 0.0001	
D^2	1.44E+07	1	1.44E+07	479.71	< 0.0001	
Residual	4.21E+05	14	30092.55			
Lack of Fit	3.20E+05	10	31990.69	1.26	0.4438	not significant
Pure Error	1.01E+05	4	25347.2			
Cor Total	1.03E+08	28				

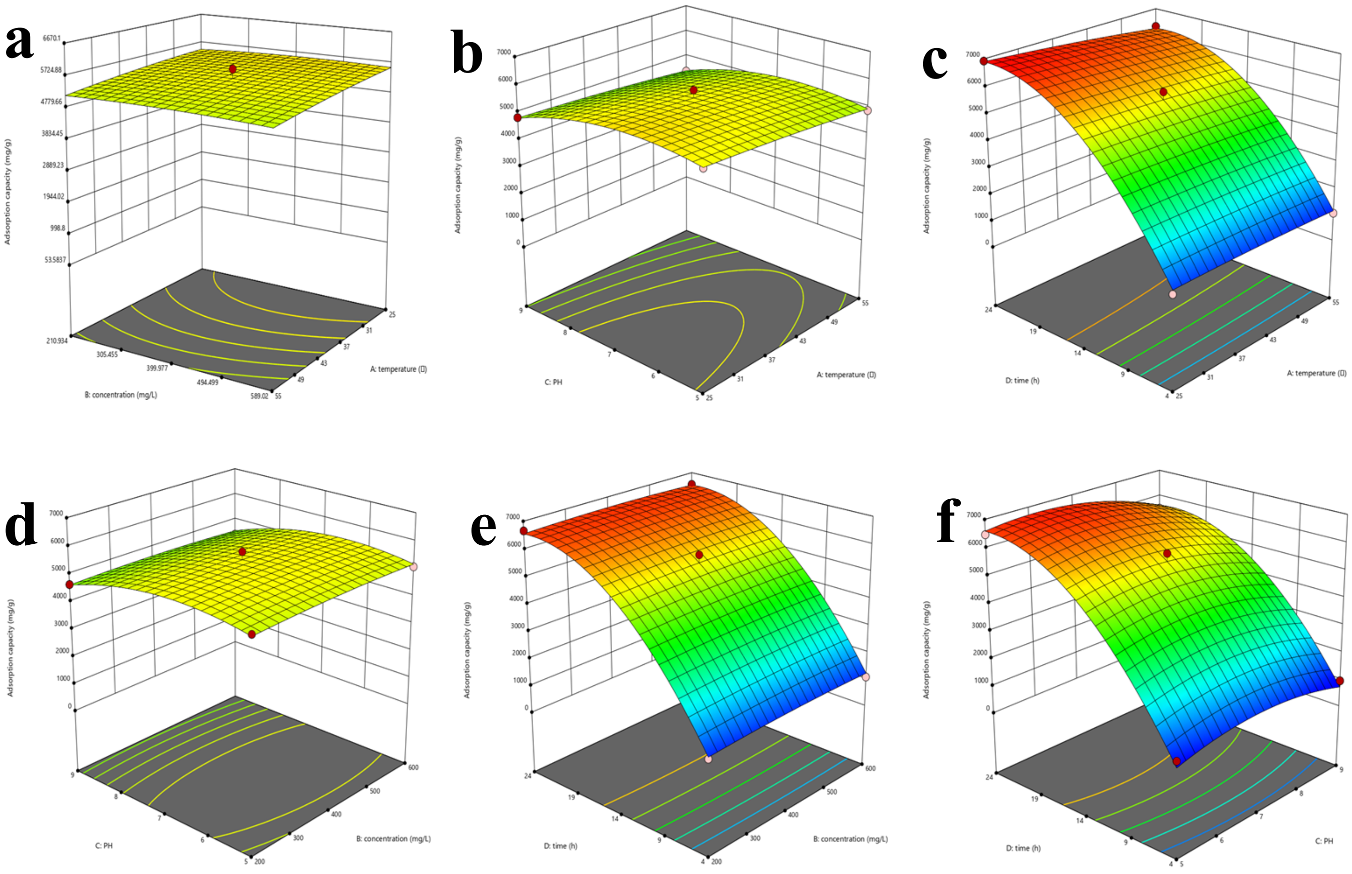

Fig. S1. 3D response surface plots for the combined effects of variables (a) temperature (A) - initial concentration (B), (b) temperature (A) – pH (C), (c)

temperature (A) – time (D), (d) initial concentration (B) - pH (C), (e) initial concentration (B) – time (D) and (f) pH (C) – time (D) on the response.

To understand the main effects and combined effects of these two variables on the response according to the three-dimensional response surface plot. Fig S1a shows the interaction between variables A and B on the adsorption of CR. For any given value of variable A, as the value of variable B increases, a continuous increasing trend in the adsorption capacity can be observed. This may be because the greater the concentration of the solution, the stronger the driving force for adsorption. The maximum adsorption capacity of 5719 mg/g can be achieved when using a CR solution with a concentration of 600 mg/L.

Fig S1b shows the interaction between A and C on CR. When variable A remains unchanged, the adsorption capacity first increases and then decreases as the pH (C) increases. The maximum adsorption capacity of 5849 is reached when the pH is 7. Fig S1c shows the interaction between variables A and D on the adsorption of CR. Given that A remains unchanged, as the time (D) increases, the adsorption capacity also continuously increases. When the adsorption time is 24 h, the maximum adsorption capacity can reach 6924 mg/g. This is because as the adsorption time prolongs, the adsorbate can reach saturation, and thus the adsorption capacity becomes larger.

Fig S1d shows the interaction between B and C on the adsorption of CR. As B increases, for any given value of C, the adsorption capacity will increase, and the maximum adsorption capacity of 5849 mg/g is reached when the pH is 7.

Fig S1e shows the interaction between B and D on the adsorption of CR. Given that the value of B remains unchanged, as the value of D continuously increases, the adsorption capacity will also continuously increase. When D is 24 h, the adsorption capacity reaches the maximum value of 6662 mg/g.

In addition, the response surface generated for the combined effect of variables C and D (Fig S1f) indicates that under any specific condition of variable C, the adsorption

capacity will increase as variable D increases, and the maximum adsorption capacity of 6752 mg/g is reached at 24 h.

Notes and references

1 N. Rahman and P. Varshney, *RSC Adv.*, 2020, **10**, 20322–20337.

2 N. Rahman and A. Raheem, *J. Mol. Liq.*, 2022, **354**, 118899.

3 N. Rahman and I. Ahmad, *J. Hazard. Mater.*, 2023, **457**, 131783.

4 A. Asfaram, M. Ghaedi, K. Dashtian and G. R. Ghezelbash, *ACS Sustain. Chem. Eng.*, 2018, **6**, 4549–4563.

5 N. Rahman and U. Haseen, *Ind. Eng. Chem. Res.*, 2014, **53**, 8198–8207.

6 N. Rahman and I. Ahmad, *J. Hazard. Mater.*, 2023, **457**, 131783.
